# A Novel *Staphylococcus* Podophage Encodes a Unique Lysin with Unusual Modular Design

**DOI:** 10.1128/mSphere.00040-17

**Published:** 2017-03-22

**Authors:** Katie Cater, Vidya Sree Dandu, S. M. Nayeemul Bari, Kim Lackey, Gabriel F. K. Everett, Asma Hatoum-Aslan

**Affiliations:** a Department of Biological Sciences, The University of Alabama, Tuscaloosa, Alabama, USA; b Center for Phage Technology, Texas A&M University and Texas A&M AgriLife, College Station, Texas, USA; University of Nebraska Medical Center

**Keywords:** *Staphylococcus*, antimicrobial agents, bacteriophage lysis, bacteriophage therapy, bacteriophages

## Abstract

Drug-resistant staphylococci, particularly *Staphylococcus aureus* and *Staphylococcus epidermidis*, are leading causes of hospital-acquired infections. Bacteriophages and their peptidoglycan hydrolytic enzymes (lysins) are currently being explored as alternatives to conventional antibiotics; however, only a limited diversity of staphylococcal phages and their lysins has yet been characterized. Here, we describe a novel staphylococcal phage and its lysins. Bacteriophage Andhra is the first reported *S. epidermidis* phage belonging to the family *Podoviridae*. Andhra possesses an 18,546-nucleotide genome with 20 open reading frames. BLASTp searches revealed that gene product 10 (gp10) and gp14 harbor putative catalytic domains with predicted peptidase and amidase activities, characteristic functions of phage lysins. We purified these proteins and show that both Andhra_gp10 and Andhra_gp14 inhibit growth and degrade cell walls of diverse staphylococci, with Andhra_gp10 exhibiting more robust activity against the panel of cell wall substrates tested. Site-directed mutagenesis of its predicted catalytic residues abrogated the activity of Andhra_gp10, consistent with the presence of a catalytic CHAP domain on its C terminus. The active site location combined with the absence of an SH3b cell wall binding domain distinguishes Andhra_gp10 from the majority of staphylococcal lysins characterized to date. Importantly, close homologs of Andhra_gp10 are present in related staphylococcal podophages, and we propose that these constitute a new class of phage-encoded lysins. Altogether, our results reveal insights into the biology of a rare family of staphylococcal phages while adding to the arsenal of antimicrobials with potential for therapeutic use.

**IMPORTANCE** The spread of antibiotic resistance among bacterial pathogens is inciting a global public health crisis. Drug-resistant *Staphylococcus* species, especially *S. aureus* and *S. epidermidis*, have emerged in both hospital and community settings, underscoring the urgent need for new strategies to combat staphylococcal infections. Bacterial viruses (phages) and the enzymes that they use to degrade bacterial cell walls (lysins) show promise as alternative antimicrobials; however, only a limited variety of staphylococcal phages and their lysins have yet been identified. Here, we report the discovery and characterization of a novel staphylococcal phage, Andhra. We show that Andhra encodes two lysins (Andhra_gp10 and Andhra_gp14) that inhibit growth and degrade the cell walls of diverse staphylococci, including *S. aureus* and *S. epidermidis* strains. Andhra and its unique lysins add to the arsenal of antimicrobials with potential for therapeutic use.

## Abstract (precis)

The spread of antibiotic resistance among bacterial pathogens is inciting a global public health crisis. Drug-resistant *Staphylococcus* species, especially *S. aureus* and *S. epidermidis*, have emerged in both hospital and community settings, underscoring the urgent need for new strategies to combat staphylococcal infections. Bacterial viruses (phages) and the enzymes that they use to degrade bacterial cell walls (lysins) show promise as alternative antimicrobials; however, only a limited variety of staphylococcal phages and their lysins have yet been identified. Here, we report the discovery and characterization of a novel staphylococcal phage, Andhra. We show that Andhra encodes two lysins (Andhra_gp10 and Andhra_gp14) that inhibit growth and degrade the cell walls of diverse staphylococci, including *S. aureus* and *S. epidermidis* strains. Andhra and its unique lysins add to the arsenal of antimicrobials with potential for therapeutic use.

## OBSERVATION

Pathogenic staphylococci resistant to multiple antibiotics continue to impose a heavy burden on global public health (1). *Staphylococcus aureus* causes moderate to fatal infections in a variety of body sites (2), and asymptomatic nasal carriage in over 25% of the population constitutes a major public health risk (3, 4). *Staphylococcus epidermidis* is a ubiquitous skin commensal and opportunistic pathogen that is responsible for the majority of infections associated with indwelling medical devices (5). To compound the high frequency of antibiotic resistance already observed in many clinical *S. epidermidis* isolates, these organisms have a propensity to form biofilms, which aid in surface colonization and provide enhanced tolerance to antibiotics (5). Drug-resistant staphylococci are a leading cause of hospital-acquired infections (6) and have more recently emerged in community settings (7), underscoring the urgent need for alternative approaches to combat staphylococcal infections.

Phages and their peptidoglycan hydrolytic enzymes (lysins) show significant promise as alternative treatments of staphylococcal infections (8–10). Both phages and their lysins have several advantages over conventional antibiotics, including their ability to (i) kill bacteria that are resistant to antibiotics, (ii) target a narrow spectrum of bacterial pathogens while leaving beneficial microbes unharmed, and (iii) penetrate and dissipate biofilms. All known staphylococcal phages have tubular tails, icosahedral heads, and double-stranded DNA genomes (11, 12). Based on these features, they have been classified into three morphological families with characteristic genome lengths: *Podoviridae* (<20 kb), *Siphoviridae* (~40 kb), and *Myoviridae* (>125 kb). Of the ~200 staphylococcal phages reported, the majority (~71%) are temperate with siphophage morphology and a host range limited to *S. aureus* (12). Temperate phages can form prophages carrying pathogenesis factors and can promote fitness and pathogenicity through the mobilization of virulence factors and pathogenicity islands (13). Thus, the staphylococcal siphophages are unsuitable for whole-phage therapy. Obligatorily lytic phages that attack staphylococci belong to the families *Myoviridae* and *Podoviridae*, of which myophages are more common and show promise as whole-phage therapeutics (8, 9). However, phages are replete with uncharacterized genes, which could cause unexpected downstream side effects. Further, regulatory constraints in Europe and the United States present challenges to the therapeutic use of phages in humans (14). As more viable alternatives, purified phage-encoded lysins have been shown to kill drug-resistant staphylococci *in vitro* and in animal models (8–10, 15). Moreover, many of these lysins work synergistically with small-molecule antibiotics and are considered less likely to promote the evolution of bacterial resistance (10). Since the use of cocktails of antimicrobials with a variety of targets and multiple modes of action can more effectively limit the emergence of resistant bacterial strains, we sought to expand the growing arsenal of staphylococcal phages and their lysins.

## 

### Phage isolation.

**FIG 1:**
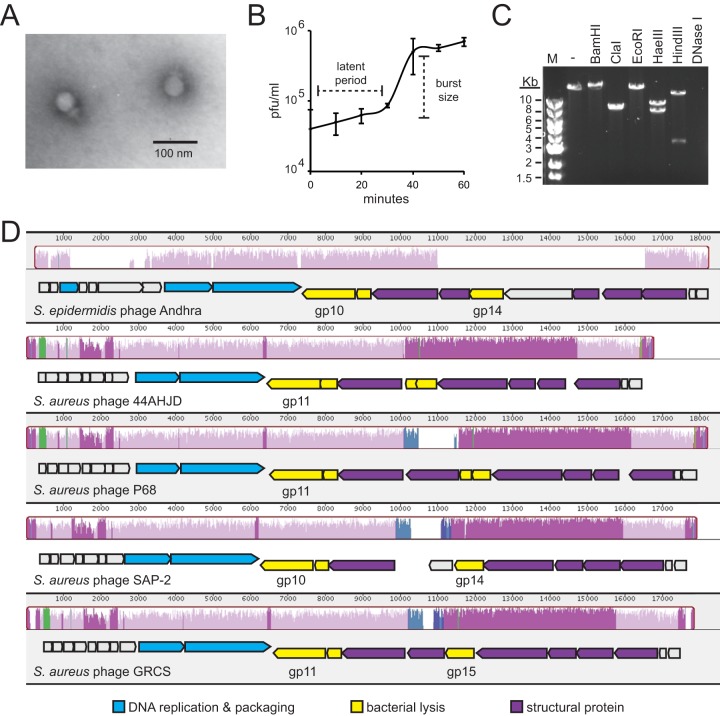
Phenotypic and genotypic features of bacteriophage Andhra. (A) Andhra was stained with uranyl acetate and imaged using transmission electron microscopy at ×200,000 magnification. The morphology is consistent with *Podoviridae*. (B) A one-step growth curve is shown. *S. epidermidis* RP62a was challenged with phage Andhra, and PFU were enumerated at 10-min intervals. An average of triplicate measurements is shown. The latent period is defined as the time between adsorption and the beginning of the first burst, and the burst size is defined as the ratio of final plaque count to the initial count. (C) Andhra genomic DNA was extracted, subjected to digestion by the indicated enzymes, and resolved on a 1% agarose gel. Lane M, 1-kb DNA marker; lane -, no enzyme added. (D) A whole-genome sequence alignment of *S. epidermidis* phage Andhra with indicated *S. aureus* podophages was generated using the Mauve software (http://darlinglab.org/mauve/mauve.html). Homologous genome regions are indicated with similarly colored histograms, and regions that lack detectible homology remain uncolored. Below the similarity histograms, open reading frames are indicated by block arrows pointing in the direction of transcription. Specific gene products that are discussed in the text are labeled underneath each open reading frame. BLASTp was used to assign putative functions for phage Andhra genes, and the GenBank annotations were used for gene function assignments for the other podophages. Gene products with related functions are similarly colored according to the key at the bottom.

Since fewer than 10 *S. epidermidis* phages have been reported thus far (12), and because the clinical isolate RP62a possesses multiple phage defense mechanisms, including a functional CRISPR-Cas (clustered regularly interspaced short palindromic repeat) system (16), we used this organism as a host to discover phages capable of subverting such defenses. Phage Andhra was captured by coculturing *S. epidermidis* RP62a with raw sewage and purified by replating individual plaques several times with this host. Transmission electron microscopy revealed that Andhra possesses a morphology consistent with members of the rare lytic family *Podoviridae* within the genus *P68like* virus (Fig. 1A; see also Table S1 in the supplemental material).

10.1128/mSphere.00040-17.4 TABLE S1  General features of podophage Andhra. Download TABLE S1, DOCX file, 0.1 MB. Copyright © 2017 Cater et al. 2017 Cater et al. This content is distributed under the terms of the Creative Commons Attribution 4.0 International license.

### Phenotypic features.

Andhra has a latent period of approximately 30 min and a burst size of 9.3 ± 2.4 phage particles released per infected cell (Fig. 1B). While plaque formation is readily apparent on its original host, Andhra causes lysis from without (LO) on related staphylococci (Table S2). LO is characterized by a general restriction of growth at the site of phage application on a bacterial lawn, without formation of plaques (17). A variety of explanations could account for this phenomenon, including the activity of phage-encoded peptidoglycan hydrolytic enzymes (tail-associated and/or free-floating lysins) present in the phage preparation. The possibility of broad-spectrum activity in its lysin(s) prompted the genomic characterization of phage Andhra.

10.1128/mSphere.00040-17.5 TABLE S2  Host range of podophage Andhra. Download TABLE S2, DOCX file, 0.1 MB. Copyright © 2017 Cater et al. 2017 Cater et al. This content is distributed under the terms of the Creative Commons Attribution 4.0 International license.

### Genotypic features.

Genomic DNA from Andhra was purified and subjected to enzymatic digestion (Fig. 1C) and whole-genome sequencing. Digestion with DNase I and various restriction enzymes confirmed a double-stranded DNA genome. Whole-genome sequencing revealed that phage Andhra has an 18,546-nucleotide (nt) genome with 155-nt terminal inverted repeats and 20 predicted open reading frames (Fig. 1D; Table S3). A BLASTn search using the Megablast algorithm against the whole Andhra genome returned regions of homology in a group of *S. aureus* podophages, which share up to 71% identity over 32% of their genomes. Multiple sequence alignments revealed that Andhra is distantly related to other staphylococcal podophages (Fig. S1), sharing a central module of conserved core genes, with some unique sequences on its peripheries (Fig. 1D). Genome architectures of these phages are also conserved, with two apparent transcriptional units converging near the center. BLASTp searches provided functional assignments for 12 out of the 20 gene products in phage Andhra (Table S3). Similarly to its *S. aureus* podophage relatives, Andhra exhibits clustering of genes into related functional groups and apparent conservation of gene order (Fig. 1D).

10.1128/mSphere.00040-17.1 FIG S1  Phylogenetic tree built on whole-genome sequence alignments of staphylococcal podophages. The evolutionary history was inferred using the neighbor-joining method (N. Saitou and M. Nei, Mol Biol Evol 4:406–425, 1987). The optimal tree with the sum of branch lengths of 0.78589941 is shown. The tree is drawn to scale, with branch lengths in the same units as those of the evolutionary distances used to infer the phylogenetic tree. The evolutionary distances were computed using the maximum composite likelihood method (K. Tamura, M. Nei, and S. Kumar, Proc Natl Acad Sci U S A 101:11030–11035, 2004, https://doi.org/10.1073/pnas.0404206101) and are in units of the number of base substitutions per site. The analysis involved 10 nucleotide sequences. Codon positions included were 1st+2nd+3rd+Noncoding. All positions containing gaps and missing data were eliminated. There were a total of 14,929 positions in the final data set. Evolutionary analyses were conducted in MEGA7 (S. Kumar, G. Stecher, and K. Tamura, Mol Biol Evol 33:1870–1874, 2016, https://doi.org/10.1093/molbev/msw054). Download FIG S1, EPS file, 1.5 MB. Copyright © 2017 Cater et al. 2017 Cater et al. This content is distributed under the terms of the Creative Commons Attribution 4.0 International license.

10.1128/mSphere.00040-17.6 TABLE S3  Phage Andhra predicted gene products and putative functions. Download TABLE S3, DOCX file, 0.1 MB. Copyright © 2017 Cater et al. 2017 Cater et al. This content is distributed under the terms of the Creative Commons Attribution 4.0 International license.

### Lysins of phage Andhra.

BLASTp results predict that gene products 10 and 14 (Andhra_gp10 and Andhra_gp14, respectively) possess catalytic domains with characteristic functions of lysins: Andhra_gp10 harbors a C-terminal CHAP domain with putative peptidase activity, and Andhra_gp14 possesses a putative Zn-binding amidase catalytic site on its N terminus. To date, only two staphylococcal podophage lysins have been characterized *in vitro*: SAL-2 (18) and PlyGRCS (19), which are encoded by *S. aureus* podophages SAP-2 and GRCS, respectively. For simplicity, these will be referred to as SAP-2_gp14 and GRCS_gp15, respectively (Fig. 1D). While Andhra_gp14 is encoded in an analogous genomic position, just upstream of phage tail genes (Table S3), Andhra_gp14 shares little or no homology with SAP-2_gp14 and GRCS_gp15 (~18% identity according to a Clustal Omega alignment [Fig. S2A]). In contrast, Andhra_gp10 is located in the conserved core region found in other staphylococcal podophages (Fig. 1D) and shares 68% identity with SAP-2_gp10 and GRCS_gp11 (Fig. S2B), as well as 44AHJD_gp11 and P68_gp11, hypothetical proteins from 44AHJD and P68, the prototypical *S. aureus* podophages. Thus far, none of these proteins have been characterized *in vitro*.

10.1128/mSphere.00040-17.2 FIG S2  Sequence alignments of podophage proteins. (A) Alignments of Andhra_gp14 with analogous proteins SAP-2_gp14 and GRCS_gp15. (B) Alignments of Andhra_gp10 with homologous proteins SAP-2_gp10 and GRCS_gp11. Conserved residues are highlighted in red, the entire predicted CHAP domain is underlined, and asterisks indicate the conserved cysteine and histidine residues of the predicted CHAP domain in Andhra_gp10. Alignments were generated using the Clustal Omega algorithm, and images were created using ESPript 3.0 (http://espript.ibcp.fr/ESPript/ESPript/). Download FIG S2, PDF file, 1.7 MB. Copyright © 2017 Cater et al. 2017 Cater et al. This content is distributed under the terms of the Creative Commons Attribution 4.0 International license.

**FIG 2:**
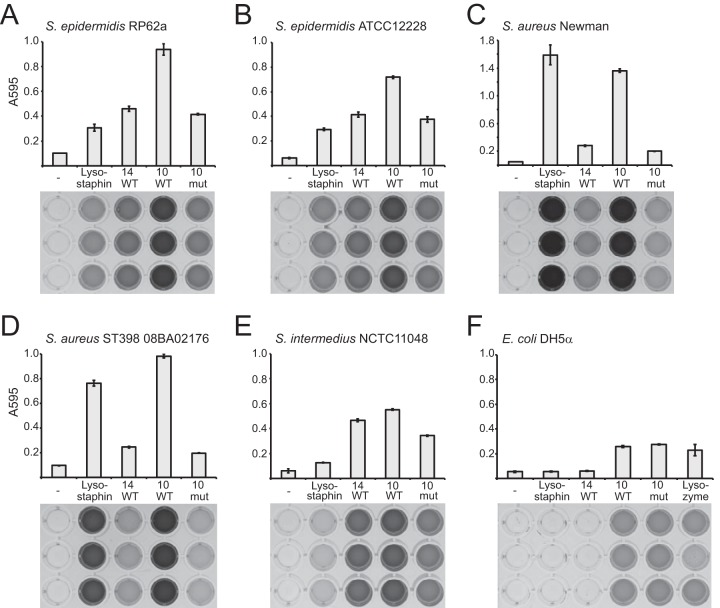
Cell wall hydrolytic activities of Andhra lysins. A dye release assay was used to quantify the cell wall hydrolytic activity of Andhra_gp14 (14 WT), Andhra_gp10 (10 WT), Andhra_gp10^C354A,H420A^ (10 mut), lysostaphin, or lysozyme. Cell walls from indicated bacterial strains were labeled with Remazol brilliant blue dye and used as the substrates. Enzymes (3 μg) or IMAC buffer (-) was incubated with an excess of labeled cell wall substrate at 37°C for 3 h. The dye released into the supernatant was quantified by measuring the optical density of the soluble fraction at 595 nm. The experiment was conducted in triplicate, and average measurements are shown (top). Images of 96-well plates containing soluble fractions corresponding to each measurement are also shown (bottom).

To test their *in vitro* activities, recombinant Andhra_gp14 and Andhra_gp10 were overexpressed in *Escherichia coli* and purified (Fig. S3A). When combined with live cells, both proteins inhibited the growth of *S. epidermidis* and *Staphylococcus intermedius* strains, with little or no effect on *S. aureus* growth (Fig. S3B). To more directly quantify their ability to degrade cell wall substrates derived from these organisms, a dye-release assay was used (Fig. 2). In this assay, heat-killed bacterial cells are labeled with Remazol brilliant blue dye, which covalently links to insoluble cell walls and is released into solution upon cell wall degradation (20). We observed that both Andhra_gp14 and Andhra_gp10 degrade the cell walls of these diverse staphylococci, including *S. aureus* strains (Fig. 2A to E). Andhra_gp10 exhibited a more robust activity against the tested strains, with moderate ability to degrade even *E. coli* cell walls (Fig. 2F).

10.1128/mSphere.00040-17.3 FIG S3  Bacterial growth inhibition by purified recombinant lysins. (A) Andhra_gp14 (14 WT), Andhra_gp10 (10 WT), and Andhra_gp10^C354A, H420A^ (10 mut) were overexpressed on pET28b-based constructs in *E. coli* and purified. Proteins were resolved by SDS-PAGE and visualized using Coomassie G-250 staining. The dotted line separates data derived from two separate gels. (B) Indicated bacterial strains were mixed with purified proteins (3 μg) or IMAC buffer and grown overnight at 37°C. Shown is an average of triplicate optical density (OD_600_) measurements following 12 to 16 h of growth. Representative data for at least two independent trials are shown. Download FIG S3, EPS file, 2.7 MB. Copyright © 2017 Cater et al. 2017 Cater et al. This content is distributed under the terms of the Creative Commons Attribution 4.0 International license.

With few exceptions, the majority of characterized staphylococcal lysins possess an N-terminal active site(s) and C-terminal SH3b cell wall binding domains (15, 21). Andhra_gp10 lacks a detectable SH3b domain and instead possesses a predicted active site on its C terminus. To confirm the active site location, the conserved cysteine (C354) and histidine (H420) residues of the predicted CHAP domain were changed to alanines (Fig. S2B), and this mutant variant was purified (Fig. S3A) and tested for activity *in vitro* (Fig. 2). While its activity against staphylococcal cell walls was severely impaired, Andhra_gp10^C354A,H420A^ exhibited an activity comparable to that of the wild type against *E. coli* cell walls. These observations are consistent with the presence of a C-terminal CHAP domain and suggest the possibility of a second distinct active site in Andhra_gp10, a rare feature of some staphylococcal lysins (15).

### Conclusions.

Here, we provide the first report of a *Podoviridae S. epidermidis* phage and demonstrate growth inhibition and cell wall hydrolytic activities of two of its enzymes: Andhra_gp14 and Andhra_gp10. Andhra_gp14 exhibits moderate hydrolytic activity against staphylococcal cell walls and lacks homologs in podophages specific for *S. aureus*. These observations, combined with its genomic position adjacent to genes encoding minor and major tail proteins, support our speculation that Andhra_gp14 is a tail-associated lysin that aids in adsorption of phage DNA during initial stages of infection. In contrast, Andhra_gp10 is encoded just downstream of a putative holin (a membrane-degrading enzyme) and exhibits robust hydrolytic activity against the strains tested, suggesting that this protein is the main lysin used to degrade cell walls from within the host and facilitate virion escape at the end of the replication cycle. Andhra_gp10 has a C-terminal CHAP domain and lacks an SH3b cell wall binding domain, two distinguishing features that make this protein a unique and important addition to the growing collection of phage-encoded lysins. Homologs of Andhra_gp10 are present in other staphylococcal podophages, and we therefore propose that these enzymes represent a new class of phage-encoded lysins. Altogether, our results reveal insights into the biology of a rare family of staphylococcal phages while adding to the arsenal of antimicrobials with potential for therapeutic use.

### Strains and growth conditions.

All staphylococcal strains used in this study were a generous gift from L. A. Marraffini. Staphylococcal strains were cultured in tryptic soy broth (TSB; Difco). *E. coli* DH5α and BL21(DE3) Codon Plus cells (EMD Millipore) were grown in Luria-Bertani broth (LB; Difco) or Terrific broth (Amresco) for protein purification. Medium was supplemented with 30 μg/ml chloramphenicol [for selection of *E. coli* BL21(DE3)] and 50 μg/ml kanamycin (for selection of pET28b-His_10_Smt3-based constructs). For routine propagation, bacteria were grown at 37°C in an orbital shaker set to 160 rpm.

### Phage isolation and purification.

Phage Andhra was isolated from raw sewage following an enrichment protocol with three consecutive days of coculturing with *S. epidermidis* RP62a. Raw sewage was collected from the Hilliard Fletcher wastewater treatment plant in Tuscaloosa, AL; treated with chloroform (final concentration of 2%); and vortexed for 1 min. Treated sewage was clarified by centrifugation at 10,000 × *g* to remove debris. Sewage supernatant was combined with TSB (1:1) and CaCl_2_ (5 mM). An overnight culture of *S. epidermidis* RP62a was diluted into this mixture (1:100) and incubated overnight at 37°C with agitation. The culture was pelleted at 3,000 × *g*, and the resulting supernatant was passed through a 0.2-μm filter. The filtrate was combined 1:1 with fresh TSB and cocultured with *S. epidermidis* RP62a twice more as described above. The lysate recovered after the third overnight coculture was assayed for the presence of plaques using a double-agar overlay method as follows: heart infusion agar (HIA; Hardee Diagnostics) prepared at half the recommended concentration was equilibrated to 55°C. Equilibrated HIA plus CaCl_2_ (5 mM) was combined with an overnight culture of *S. epidermidis* RP62a (at a 1:100 final dilution), and 4 ml of this mixture was overlaid atop tryptic soy agar (TSA) plates containing 5 mM CaCl_2_. Tenfold dilutions of lysate were spotted atop the semisolid HIA layer, allowed to air dry, and incubated overnight at 37°C. A well-separated plaque was picked with a sterile pipette tip, combined with 1 ml of TSB, and vortexed at high speed for 1 min. The phage suspension was filtered and spotted on semisolid agar as described above. Individual plaques were picked and replated at least five times to obtain a purified phage preparation.

### High-titer phage cultivation.

High-titer phage lysates for host range analysis and DNA extraction were prepared by combining 5 to 10 purified plaques into 1 ml of TSB and vortexing for 1 min. The resulting phage suspension was passed through a 0.2-μm filter and added to 50 ml of fresh mid-log *S. epidermidis* RP62a cells grown in TSB plus CaCl_2_ (5 mM). Cultures were incubated for 3 to 4 h at 37°C in an orbital shaker until clearing was observed. Lysates were pelleted to remove cell debris, and resulting supernatant was passed through a 0.2-μm filter. Phage titers were determined by spotting 10-fold dilutions of lysate on semisolid agar as described above. Following overnight incubation at 37°C, plaques were enumerated and titers were determined as PFU per milliliter.

### Transmission electron microscopy.

Phages from 1 ml of high-titer lysate (at least 1 × 10^9^ PFU/ml) were pelleted using high-speed centrifugation (1 h at 24,000 × *g*) and washed three times with 1 ml of a 0.1 M ammonium acetate solution. After the third wash, phage pellets were resuspended in a final volume of 50 μl. Five microliters of phage suspension was applied to a Formvar-coated copper grid and allowed to settle for 5 min. Excess liquid was wicked away, and phages were stained for 30 s with 2% uranyl acetate. Grids were air dried and visualized using a Hitachi 7650 transmission electron microscope located at the University of Alabama Optical Analysis Facility.

### One-step growth curve.

The one-step growth curve was determined as described in reference 22 with minor modifications. Briefly, 20 ml of mid-log *S. epidermidis* RP62a cells was combined with phage lysate to achieve a multiplicity of infection (MOI) of 0.1. Phages were allowed to adsorb to the host for 10 min at 37°C without agitation. Cells were pelleted by centrifugation at 8,000 × *g* for 5 min, washed once with 20 ml of TSB, and resuspended in 20 ml of fresh TSB. Cells were incubated at 37°C, 200-μl samples were taken at 10-min intervals and pelleted, and supernatant titers were determined immediately as described above. The experiment was carried out in triplicate. The latent period was defined as the time period from adsorption to the beginning of the first burst, and the burst size was calculated as the ratio of plaques observed after the first burst to that prior to the first burst.

### Genomic DNA extraction.

Phage DNA was extracted as described previously (23). Briefly, 20 ml of high-titer phage lysate (≥1 × 10^9^ PFU/ml) was incubated with DNase I and RNase A (10 μg/ml of each) for 30 min at 37°C. Ten milliliters of precipitant solution (30% [wt/vol] polyethylene glycol [PEG] 8000 and 3 M NaCl) was combined with the lysate and incubated at 4°C overnight. Phages were pelleted by centrifugation for 10 min at 10,000 × *g* and 4°C. The supernatant was carefully decanted, and phage pellet was resuspended in 500 μl of resuspension buffer (5 mM MgSO_4_, 10 mM EDTA). The phage suspension was incubated with proteinase K (100 μg/ml) at 50°C for 30 min. The phage suspension was allowed to cool to room temperature and then combined with the resin contained in the Promega Wizard DNA cleanup kit (catalog no. A7280). The phage suspension plus resin was inverted several times and applied to a minicolumn contained within the kit. The resin retained in the column was washed with 2 ml of 80% isopropanol and dried by centrifugation at 13,000 × *g* for 2 min, and DNA was eluted from the resin with 100 μl of distilled water (dH_2_O) preheated to 80°C. The concentration of recovered DNA was determined using a NanoDrop spectrophotometer.

### Genomic DNA sequencing.

DNA was sequenced in an Illumina MiSeq 250-bp paired-end run with a 550-bp insert library at the Genomic Sequencing and Analysis Facility at the University of Texas (Austin, TX). Quality-controlled trimmed reads were assembled to a single contig at 136.7-fold coverage. The contig was confirmed to be complete by PCR using primers that face the upstream and downstream ends of the DNA. Genes were predicted using SnapGene software.

### Plasmid construction. 

pET28b-His_10_Smt3-based plasmids expressing Andhra_gp10, Andhra_gp10^C354A,H420A^, and Andhra_gp14 were constructed using Gibson assembly with the primers shown in Table S4. Plasmid pET28b-His_10_Smt3 was used as a PCR template for the backbone constructs containing the wild-type proteins, Andhra genomic DNA was used to amplify the corresponding wild-type phage genes, and pET28b-His_10_Smt3-Andhra_gp10 was used as the template to create the construct containing the Andhra_gp10^C354A,H420A^ mutant. PCR products were purified using the EZNA Cycle Pure kit (Omega) and Gibson assembled, and resulting plasmids were transformed into *E. coli* DH5α. Transformants were isolated, and the constructs were purified and sequenced with primers T7T and T7P (Table S4). Confirmed constructs were then transformed into *E. coli* BL21(DE3) Codon Plus cells for protein purification.

10.1128/mSphere.00040-17.7 TABLE S4  DNA oligonucleotides used in this study. Download TABLE S4, DOCX file, 0.1 MB. Copyright © 2017 Cater et al. 2017 Cater et al. This content is distributed under the terms of the Creative Commons Attribution 4.0 International license.

### Purification of recombinant Andhra_gp14, Andhra_gp10, and Andhra_gp10^C354A,H420A^ from *E. coli*. 

Recombinant proteins were overexpressed and purified from *E. coli* BL21(DE3) Codon Plus cells containing the corresponding pET28b overexpression constructs as previously described (24) with minor modifications: the pH of all buffers was adjusted to 6.8 for the purification of Andhra_gp10 and Andhra_gp10^C354A,H420A^ and to 7.5 for the purification of Andhra_gp14. Additionally, the final dialysis for all proteins was carried out in IMAC buffer (50 mM Tris-HCl, pH 7.5, 250 mM NaCl, 10% glycerol, 50 mM imidazole). Final protein concentration was determined using the Bradford reagent (Bio-Rad), and purity was determined by resolution on a 12% SDS-PAGE gel. Proteins were stored at −20°C in IMAC buffer.

### Growth inhibition assay.

Overnight staphylococcal cultures were diluted 1:100 in double-strength TSB plus 1 mM MgCl_2_. Diluted cultures were distributed into a 96-well plate (50 μl per well), and purified protein (3 μg) or IMAC buffer was added into triplicate wells. The final protein concentration was 40 μg/ml. Cells were grown overnight at 37°C with agitation, and optical density measurements at 600 nm (OD_600_) were taken at 15-min intervals in a SpectraMax plate reader (Molecular Devices).

### Dye release assay.

Cell wall substrates derived from indicated bacterial strains were prepared and subjected to degradation with phage lysins as described in reference 20 with minor modifications. To prepare cell wall substrates, cells (500 ml) were grown to mid-exponential phase in TSB and autoclaved in a liquid cycle set to 35 min, and heat-killed cells were washed once with phosphate-buffered saline (PBS). The cell pellet was weighed and mixed with a Remazol brilliant blue solution (200 mM Remazol Brilliant Blue R dye [Sigma] prepared in 250 mM NaOH) at a concentration of 0.5 g cell pellet in 30 ml of solution. Mixtures were incubated at 37°C for 6 h with agitation and then transferred to 4°C for 12 h with gentle rocking. Cell wall substrate was centrifuged at 3,000 × *g* for 30 min, the supernatant was poured off, and the pellet was washed with 40 ml dH_2_O. Cell wall substrate was pelleted and washed as described above repeatedly (~5 times) until the supernatant became clear and the only coloration in the tube remained in the cell pellet. To perform the dye release assay, labeled substrate pellets were resuspended in IMAC buffer plus 1 mM MgCl_2_. The volume was adjusted until an optical density (OD) of 2.0 at 595 nm was achieved. Reaction mixtures were prepared by combining 200 μl of substrate suspension with 3 μg of enzyme (lysostaphin [Ambi Products], lysozyme [Amresco], Andhra_gp10, Andhra_gp10^C354A,H420A^, or Andhra_gp14). Triplicate reaction mixtures were prepared for each enzyme treatment and incubated at 37°C for 3 h, with occasional inversion of tubes (once per hour). Reactions were stopped with the addition of 25 μl of ethanol, and cell wall debris was pelleted by centrifugation at 13,000 × *g* for 5 min. One hundred microliters of supernatant from each reaction mixture was placed into a 96-well flat-bottom plate. Plates were imaged and read for OD_595_.

### Accession number(s).

The assembled whole-genome sequence of phage Andhra was deposited in GenBank under accession no. KY442063.
