# Supplementary material for: A Novel Staphylococcus Podophage Encodes a Unique Lysin with Unusual Modular Design
Source: mSphere. 2017 Mar 22;2(2):e00040-17. doi: 10.1128/mSphere.00040-17 (PMC5362749; doi:10.1128/mSphere.00040-17)
Supplement: TABLE S1 [file sph002172255st4.docx]

**Table S1.** General features of podophage Andhra.

| Host | *S. epidermidis* RP62a |
| --- | --- |
| Capsid diameter*^a^* | 42.7 ± 1.5 nm |
| Latent period | 30 minutes |
| Burst size*^b^* | 9.3 ± 2.4 |
| Genome size | 18,546 bp |
| GC content (%) | 30 |
| End features | 155 bp inverted repeats |
| Predicted no. of genes | 20 |
| Putative functions [no. (%)] | 12 (60) |
| Conserved hypothetical [no. (%)] | 2 (10) |
| No database match [no. (%)] | 6 (30) |

*a* Represents average diameter of ten phage particles measured across three axes.

*b* Calculated as the ratio of plaque forming units (pfus) per ml observed after and before the first burst in one-step growth curve (at 50 and 10 minutes, respectively, in Fig. 1B).
