# Supplementary material for: A Novel Staphylococcus Podophage Encodes a Unique Lysin with Unusual Modular Design
Source: mSphere. 2017 Mar 22;2(2):e00040-17. doi: 10.1128/mSphere.00040-17 (PMC5362749; doi:10.1128/mSphere.00040-17)
Supplement: TABLE S2 [file sph002172255st5.docx]

**Table S2.** Host range of podophage Andhra

| **Strain** | **Average pfu/mL*^a^*** |
| --- | --- |
| *Staphylococcus epidermidis* RP62a | 1.2 (± 0.3) x 10^9^ |
| *Staphylococcus epidermidis* ATCC12228 | 0 |
| *Staphylococcus epidermidis* 1457 | 0 (LO*^b^*) |
| *Staphylococcus aureus* Newman | 0 |
| *Staphylococcus aureus* ST398 08BA02176 | 0 |
| *Staphylococcus aureus* RN4220 | 0 |
| *Staphylococcus intermedius* NCTC11048 | 0 (LO) |
| *Staphylococcus pseudointermedius* ED99 | 0 (LO) |
| *Staphylococcus delphini* 8086 | 0 |

*a* Represents average of three independent replicates.

*b* Lysis from without, or slight clearing of cells in the presence of concentrated phage lysate without the appearance of plaques in diluted phage lysate.
