# Supplementary material for: A Novel Staphylococcus Podophage Encodes a Unique Lysin with Unusual Modular Design
Source: mSphere. 2017 Mar 22;2(2):e00040-17. doi: 10.1128/mSphere.00040-17 (PMC5362749; doi:10.1128/mSphere.00040-17)
Supplement: TABLE S3 [file sph002172255st6.docx]

**Table S3.** Phage Andhra predicted gene products and putative functions.

| Gene Product | Theoretical  Mass (kDa) | Predicted function (predicted functional domains) |
| --- | --- | --- |
| 1 | 9.4 | Conserved hypothetical*^a^* |
| 2 | 8.6 | Hypothetical*^b^* |
| 3 | 19.6 | Single-stranded DNA binding protein |
| 4 | 8.1 | Hypothetical |
| 5 | 9.4 | Hypothetical |
| 6 | 46.2 | Esterase or lipase activity (C-terminal SGNH/GDSL domain) |
| 7 | 19.9 | Hypothetical |
| 8 | 50.1 | Encapsidation protein |
| 9 | 90.4 | DNA Polymerase |
| 10 | 51.6 | Peptidase (C-terminal CHAP domain) |
| 11 | 15.5 | Holin |
| 12 | 68.4 | Major tail protein |
| 13 | 32.8 | Minor tail protein |
| 14 | 35 | Endolysin (N-terminal amidase domain) |
| 15 | 67.3 | Hypothetical |
| 16 | 28.4 | Lower collar protein |
| 17 | 39.2 | Upper collar protein |
| 18 | 46.3 | Major capsid protein |
| 19 | 7.1 | Conserved hypothetical |
| 20 | 12.3 | Hypothetical |

*a* Conserved hypothetical designation indicates the presence of at least six BLASTp hits with >50% identity over >80% of the length of the protein.

*b* Hypothetical designation indicates BLASTp search returned no significant hits.
