# Supplementary material for: A Novel Staphylococcus Podophage Encodes a Unique Lysin with Unusual Modular Design
Source: mSphere. 2017 Mar 22;2(2):e00040-17. doi: 10.1128/mSphere.00040-17 (PMC5362749; doi:10.1128/mSphere.00040-17)
Supplement: FIG S2 [file sph002172255sf2.pdf]

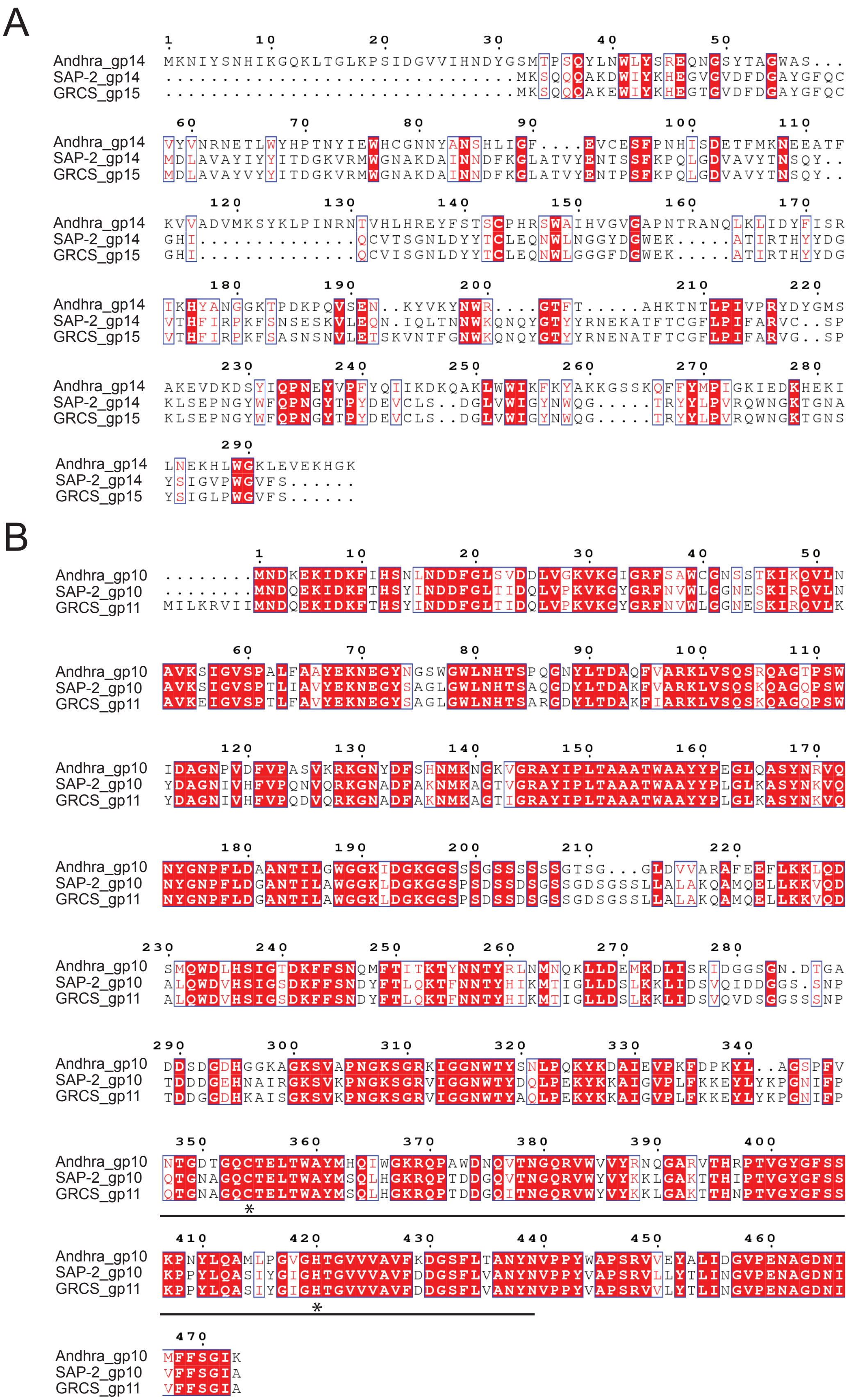

Figure S2. Cater et al.
