# Supplementary material for: A Novel Staphylococcus Podophage Encodes a Unique Lysin with Unusual Modular Design
Source: mSphere. 2017 Mar 22;2(2):e00040-17. doi: 10.1128/mSphere.00040-17 (PMC5362749; doi:10.1128/mSphere.00040-17)
Supplement: TABLE S4 [file sph002172255st7.docx]

**Table S4.** DNA oligonucleotides used in this study.

| **Primer** | **Sequence (5’-3’)** | **Purpose** |
| --- | --- | --- |
| K011 | CACAGAGAACAGATTGGTGGATCCATGAAAAACATTTATTCAAATCACA | Gibson assembly: pET28b-His_10_Smt3-*gp14* (pKC001) |
| K012 | TGTGATTTGAATAAATGTTTTTCATGGATCCACCAATCTGTTCTCTGTG |  |
| K013 | TTGAAAAGCATGGCAAGTAACTCGAGCACCAC |  |
| K014 | GTGGTGCTCGAGTTACTTGCCATGCTTTTCAA |  |
| K015 | GAGGCTCACAGAGAACAGATTGGTGGATCCATGA | Gibson assembly: pET28b-His_10_Smt3-*gp10* (pKC002) |
| K016 | CAATTTTTTCTTTATCATTCATGGATCCACCAATCTGTTCTCTGTGAG |  |
| K017 | GTGGCATTAAGTAACTCGAGCACCACCACC |  |
| K018 | GGTGGTGGTGCTCGAGTTACTTAATGCCAC |  |
| K077 | GATACTGGACAAGCTACAGAATTGACATGGGCG | Gibson assembly: pET28b-His_10_Smt3-*gp10* ^C354A,H420A^ (pKC020) |
| K078 | GTCAATTCTGTAGCTTGTCCAGTATCACCTGTATTAACAAATGG |  |
| K079 | CCGGGTGTTGGAGCTACAGGTGTTGTTGTAG |  |
| K080 | CACCTGTAGCTCCAACACCCGGTAACATCGC |  |
| T7P | TAATACGACTCACTATAGGG | Sequence confirmation for pET28b-His_10_Smt3 constructs |
| T7T | TATGCTAGTTATTGCTCAG |  |
